# Coverage of different health insurance programs and medical costs associated with chronic hepatitis C infection in mainland China: a cross-sectional survey in 20 provinces

**DOI:** 10.1186/s41124-016-0008-6

**Published:** 2016-04-28

**Authors:** Hai-yang Zhou, Shuang Liu, Su-jun Zheng, Xiao-xia Peng, Yu Chen, Carol Duan, Qing-fen Zheng, Zhao Wang, Zhong-ping Duan

**Affiliations:** 1 Wu Jieping Medical Foundation, Beijing, China; 2 Beijing Youan Hospital, Capital Medical University, Beijing, China; 3 Department of Epidemiology and Biostatistics, School of Public Health and Family Medicine, Capital Medical University, Beijing, China; 4 Department of Gastroenterology, The First Affiliated Hospital of Zhengzhou University, Zhengzhou, China

**Keywords:** Medical costs, Chronic HCV infection, Coverage of health insurance, Mainland China

## Abstract

**Background:**

Hepatitis C virus (HCV) imposes a considerable disease burden in China, with at least 10 million people chronically infected. Little is known about the financial impact of the HCV epidemic, nor about the extent to which various forms of insurance are providing HCV patients with financial protection. A cross-sectional multi-site study was conducted to acquire data that will aid policy-makers and other stakeholders in developing effective strategies to address this situation.

**Methods:**

At 29 hospitals across China, inpatients and outpatients with chronic HCV were surveyed about their insurance coverage and medical costs. Percentages, means and medians were calculated, and differences in continuous variables among multiple groups were analyzed using the Kruskal-Wallis test or Wilcoxon two-sample test.

**Results:**

Many inpatients (*N* = 593) and outpatients (*N* = 523) reported being covered by one of three major types of government health insurance, but 13 % of inpatients and 43 % of outpatients reported having no insurance. Among inpatients, the total median cost per hospitalization per patient was 8212 Renminbi (RMB). The category of expenditure with the highest median cost per hospitalization was Western medicine, followed by lab tests and Chinese medicine. The median cost per hospitalization was far higher for patients who had hepatocellular carcinoma than for those with less severe forms of liver disease. Outpatient antiviral therapy costs ranged from a median of 377 RMB for ribavirin to a median of 37,400 RMB for pegylated interferon-alpha for up to one year of treatment.

**Conclusions:**

For uninsured chronic HCV patients in China, inpatient and outpatient costs may be financially devastating. Research is needed on how different approaches to financing HCV treatment and care might improve health outcomes as well as achieve cost savings by enabling more people to be cured of HCV.

## Background

In China, chronic hepatitis C virus (HCV) infection is increasingly recognized as a public health problem that has major consequences for individuals, families and society in general. At least 10 million people in mainland China are chronically infected with HCV [1–3], and annual HCV incidence has increased considerably in recent years [4]. Chronic hepatitis C infection is one of the leading causes of cirrhosis and hepatocellular carcinoma (HCC). Usually, 10 to 15 % of HCV-infected patients are expected to develop cirrhosis, and the risk of HCC in persons with cirrhosis is approximately 2–4 % per year [5, 6]. Therefore China is thought to have a considerable burden of HCV-associated cirrhosis and HCC.

Hepatitis C is a curable disease [6, 7], and advances in HCV therapy have resulted in steadily higher cure rates. New direct-acting antivirals (DAAs) represent a major breakthrough in the treatment of HCV infection, but these drugs are not generally available in mainland China. Currently either interferon or peg-interferon (PEG-IFN) combined with ribavirin (RBV) is still the first-line HCV treatment in China [4, 5]. The use of PEG-IFN/RBV has resulted in sustained viral response rates ranging from 44 to 83 % in a number of Chinese studies [4], representing somewhat better outcomes than those observed worldwide for PEG-IFN/RBV. While PEG-IFN/RBV is much cheaper than the DAA regimens, the cost of PEG-IFN/RBV still imposes a heavy financial burden on many HCV patients in China.

The Chinese health system incorporates multiple types of insurance that offset medical costs for covered individuals, although a lack of comprehensive national data makes it difficult to determine the extent to which people are utilizing different types of insurance to meet their health needs. Major categories of insurance include the government employee program; government public program for urban areas; government public program for rural areas; commercial health insurance; and government-provided catastrophic health insurance. The government employee program, which is provided by the national government, is only for civil servants who are national or local government employees. The government public program for urban areas is provided by the national government for urban residents, and the insured individual must submit an annual co-pay. The government public program for rural areas is offered to farmers by national and local governments, and farmers also have a co-pay. Commercial health insurance, provided by private insurance companies, is available to anyone who meets the specified requirements. Catastrophic health insurance provided by the national government is a kind of supplementary insurance for basic national health insurance plans including the government employee program, government public program for urban areas, and government public program for rural areas. If the patient suffers from a life-threatening or highly debilitating disease, or if medical expenses exceed the limitation of the basic national health insurance coverage, the additional amount will be paid through catastrophic medical insurance. There are other types of insurance for populations such as military personnel and college students. Some people do not purchase any government or private health insurance, and thus are responsible for meeting all of their health care costs.

The financial impact of the HCV epidemic in China is not well known. This evidence gap threatens to undermine efforts to develop strategies that will improve outcomes for HCV patients. In this study, we conducted cross-sectional multisite surveys of outpatients and inpatients with chronic HCV infection. Our purpose was to acquire data that will guide policy-makers and other relevant stakeholders in making decisions that will contribute to reducing the burden of HCV disease in China.

## Methods

### Study population

**Fig. 1:**
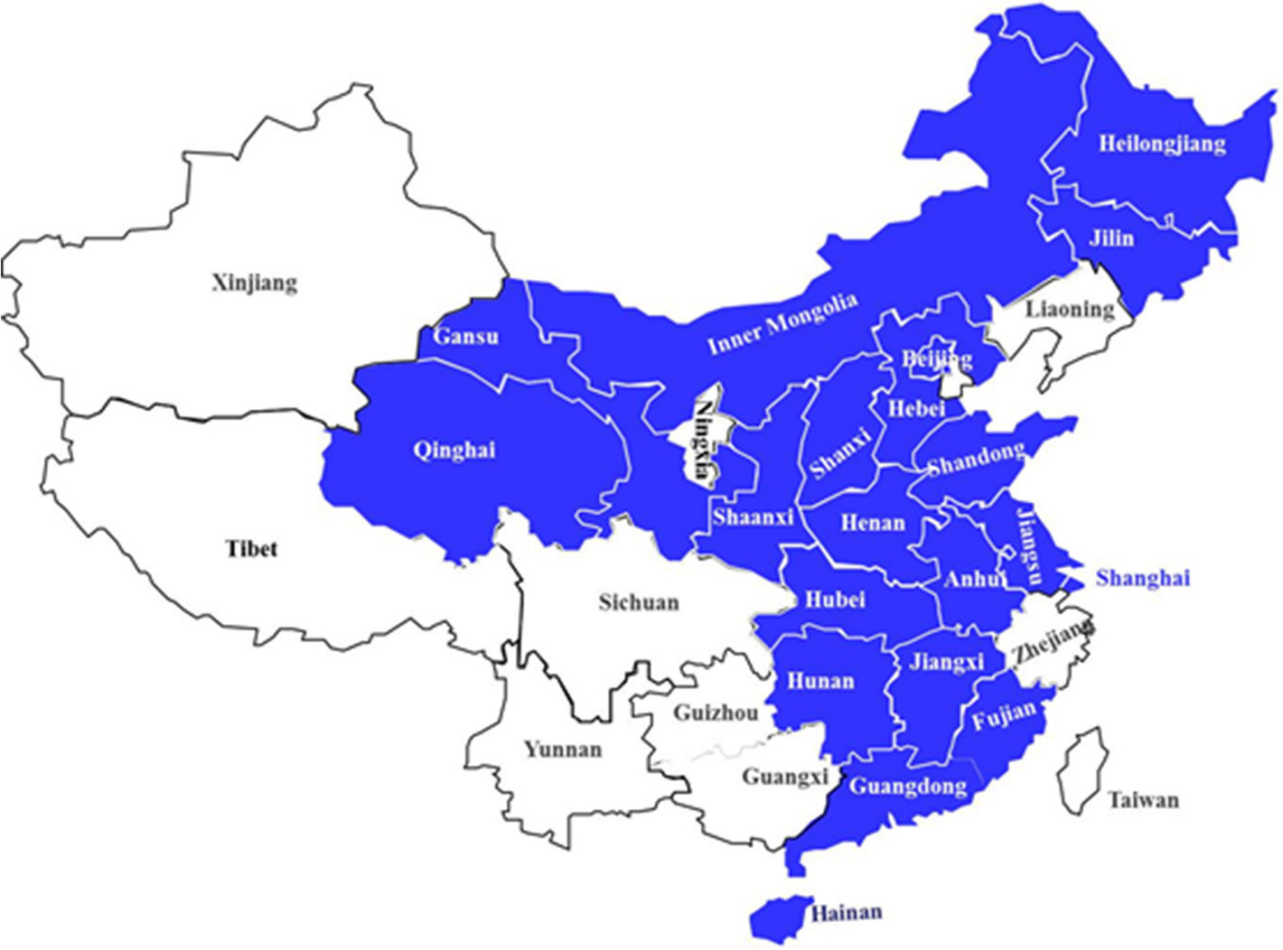
Geographic distribution of participating hospitals. Legend: Provinces with hospitals that participated in the study appear in blue

China is a very large country with many different regions and with different levels of development across regions. In an effort to reflect the overall national situation, we conducted a cross-sectional survey at 29 hospitals in 20 provinces in all regions of China (North, Northeast, East, Middle, South and Northwest) (Fig. 1). Sixteen of the 29 participating hospitals were tertiary hospitals and 13 were secondary hospitals. The 29 hospitals included 11 infectious disease specialty hospitals.

Inpatients and outpatients with a diagnosis of chronic hepatitis C were eligible to participate in the study if they were at least 18 years old and if they had tested serum-positive for anti-HCV Ab or HCV-RNA (confirmed by either HCV-RNA quantitative real-time PCR test or qualitative test) at least six months before study enrollment. Inpatients had undergone at least one hospital admission. Outpatients were only eligible to participate in the study if they had been seen for at least one year at the hospital where study enrollment was taking place. Patients were excluded from study participation if they had HIV, other forms of viral hepatitis, cancer, cardiovascular disease or other serious health conditions; if they had underlying liver disease due to other factors such as autoimmune factors, alcohol consumption or drug use; or if they were pregnant or breastfeeding. Hepatocellular carcinoma was regarded differently since it was a condition of interest for investigators, and having HCC did not disqualify patients from study participation.

### Data collection

To ensure consistency in how data were collected, the principal investigators from all study sites underwent training at Beijing Youan Hospital before study enrollment began. The training curriculum explained the inclusion and exclusion criterion, as well as introducing standard procedures for how to communicate with patients and how to instruct them to complete the study questionnaires. Each site was asked to enroll 40 to 45 individuals, including at least 20 inpatients and 20 outpatients. Sites stopped enrolling new study participants when they reached this target. Outpatients completed questionnaires in the waiting room of the outpatient department. Inpatients completed questionnaires in a quiet room in the inpatient department. Physicians provided in-person instruction to patients regarding how to complete the questionnaires. Data collection took place from 1 May to 31 December 2011. During this time, a designated clinical associate from Beijing Youan Hospital was charged with responding immediately to questions from the study sites.

Different questionnaires were used for outpatients and inpatients. Both questionnaires included the same four sections. The first section asked for the patient’s name and for demographic information such as age, sex and place of residence. The second section addressed the patient’s general condition, including the following information: the age when he or she was diagnosed with hepatitis C; insurance status; disease stage; disease complications; and time of hospitalization. The third section asked about the patient’s treatment history, including whether the patient received antiviral therapy; the names of antiviral medications; whether therapy to protect liver function was received; and whether traditional Chinese medicines were utilized. The fourth section collected information about various medical costs. Inpatients were asked about costs per hospitalization, including the cost of Western medication, traditional Chinese medicine, laboratory tests, bed charges and nursing. For HCC patients, additional questions were asked about the costs of HCC-specific treatment and HCC-related surgery. For outpatients, antiviral treatment costs were requested.

The questionnaire was designed in the Chinese language. All questions were expressed in short simple sentences such as “Where do you come from?” and “How old are you?”. Study investigators explained questions to patients who could not read. After each patient completed his or her questionnaire, the answers were checked against the patient’s medical records for accuracy. If differences were identified, the information in the medical record was used. Data management was performed by a professional statistician to ensure the soundness of the data.

Study participants were categorized according to disease severity using the following criteria. People who had chronic HCV but did not have a diagnosis of either cirrhosis or HCC were designated as “chronic HCV, no cirrhosis or HCC”. The diagnosis of chronic hepatitis C was based on the criteria used by the Chinese Society of Hepatology and the American Association for the Study of Liver Diseases (AASLD) [5, 8]. People who had been diagnosed with cirrhosis via liver biopsy or imaging but did not have a diagnosis of HCC were “chronic HCV plus cirrhosis”. People who had been diagnosed with HCC, including those with and without a diagnosis of cirrhosis, were “chronic HCV plus HCC”. The diagnosis of HCC was based on AASLD guidance and utilized radiology, AFP serology, and/or biopsy [9].

### Data analysis

Statistical analysis was performed using IBM SPSS Statistics for Windows, version 19.0 (IBM Corp, Armonk, NY, USA). Data were expressed as the mean (standard deviation), median or number (percentage). If the data had non-normal distributions, differences in continuous variables among multiple groups were analyzed using the Kruskal-Wallis Test or Wilcoxon Two-Sample Test between two groups. A two-sided p-value of <0.05 was considered statistically significant. For the comparison of inpatient costs by type of insurance, four categories were used: one for no insurance and the other three for the three types of insurance that cover nearly 85 % of Chinese citizens: government employee program, government urban public program and government rural public program [10].

### Research ethics

The study protocol was approved by the Institutional Review Board of Beijing Youan Hospital, Capital Medical University and the study was performed in accordance with the provisions of the Declaration of Helsinki 1975 and its revision. Written informed consent was obtained from all study participants. Personal information collected by investigators was only used for study purposes. Data were managed using identification numbers that were assigned to protect the privacy of study participants.

## Results

Among 1149 questionnaires submitted, 1116 (97.1 %) fulfilled data collection requirements (593 from inpatients and 523 from outpatients). Fifty-three percent of these 1116 respondents were male and 47 % were female. More than 90 % of respondents were aged 30 or older. A higher number of outpatients than inpatients were younger than 30 years, while more inpatients than outpatients were older than 60 years. An average of 18 months had elapsed from the first confirmed diagnosis of HCV infection to the time of study enrollment (Table 1).

**Table 1:** Sociodemographic characteristics of study participants

		Total patients (*n* = 1116)	Inpatients (*n* = 593)	Outpatients (*n* = 523)
Gender (%)	Female	529 (47.4)	276 (46.5)	253 (48.4)
	Male	587 (52.6)	317 (53.5)	270 (51.6)
Age, mean (standard deviation)		47.5 (13.1)	49.1 (12.8)	45.5 (13.3)
Age, median (P25, P75)		48.00 (39,57)	48.0 (40,58)	46.00 (37, 55)
Age, range (minimum, maximum)		68 (18,86)	68 (18,86)	65 (18, 83)
Age, n/%	<30	109 (9.8)	39 (6.9)	70 (13.4)
	30–39	196 (17.6)	97 (16.4)	99 (18.9)
	40–49	334 (29.9)	178 (30.0)	156 (29.8)
	50–59	278 (24.9)	153 (25.8)	125 (23.9)
	60–69	148 (13.3)	96 (16.2)	52 (9.9)
	≥70	51 (4.5)	30 (5.1)	21 (4.0)
Months elapsed since diagnosis, mean (standard deviation)		35.5 (47.9)	34.0 (47. 6)	37.4 (48.4)
Months elapsed since diagnosis, median (P25, P75)		18.0 (11.0, 38.0)	18.0 (8.0, 37.3)	22.0 (12.0,39.0)
Months elapsed since diagnosis, range (minimum, maximum)		333.0 (0.0, 333.0)	325.0 (0.0, 325.0)	333.0 (0.0, 333.0)
Region, n/%	North	201 (18.0)	109 (18.4)	92 (17.6)
	Northeast	96 (8.6)	54 (9.1)	42 (8.0)
	East	304 (27.2)	158 (26.6)	146 (27.9)
	Middle	283 (25.4)	141 (23.8)	142 (27.1)
	South	81 (7.3)	40 (6.7)	41 (7.8)
	Northwest	151 (13.5)	91 (15.3)	60 (11.5)

### Health insurance coverage

Compared with outpatients, more inpatients were covered by the government public programs for urban and rural health (46.4 % versus 30.0 % and 25.6 % versus 12.4 %, respectively). Up to 43.0 % of all outpatients reported paying for medical expenses on their own, while only 13.3 % of all inpatients reported paying on their own. (Table 2).

**Table 2:** Health insurance coverage

	Inpatients (*n* = 593)		Outpatients (*n* = 523)	
	N	(%)	N	(%)
Government employee program (total)	75	12.7	73	14.0
With catastrophic health insurance	(2)			
Government public program, urban (total)	275	46.4	157	30.0
With catastrophic health insurance	(1)		(5)	
With commercial health insurance	(1)		(2)	
Government public program, rural (total)	152	25.6	65	12.4
With commercial health insurance	(2)			
With catastrophic health insurance	(1)			
Other types of health insurance	12	2.0	3	0.6
Uninsured	79	13.3	225	43.0

### Inpatient cost per hospitalization and major categories of expenditure

The total median cost for hospitalization per time per patient was 8212 RMB. While Western medicine was the category of expenditure with the highest median cost (4701 RMB), mean costs for lab tests and Chinese medicine both exceeded 1000 RMB (Table 3). Since traditional Chinese medicine has been in existence for thousands of years, it is worth noting that the mean cost of Chinese medicine was about 8 % of the total cost of hospitalization.

**Table 3:** Inpatient costs, total and by category of expenditure (in Renminbi)

	N	Mean cost	Standard deviation	Median cost	P25	P75
Total	593	12,720	14,595	8212	5098	15,358
Western medicine	577	8255	11,192	4701	2539	9610
Lab tests	569	1574	1386	1302	709	1977
Chinese medicine	245	1038	2861	314	120	812

### Inpatient costs by type of insurance

We further analyzed inpatient medical costs per time per person by comparing inpatients with insurance coverage through the government employee program to those covered through the government urban public program and government rural public program, as well as those who were uninsured. The median total cost per hospitalization before insurance payout was 12,117 RMB for patients in the government employee program, 9535 RMB for patients in the government urban public program and 6340 RMB for patients in the government rural public program. Patients without insurance reported a median total cost per hospitalization of 6937 RMB. The differences were statistically significant (*X* ^2^ = 41.27, *p* < 0.0001) (Table 4).

**Table 4:** Inpatient costs by type of insurance (in Renminbi)

	N	Median cost	P25	P75	*X* ^2^	*P* value*
Government employee program	67^a^	12,117	7531	17,989	41.27	<0.0001
Government urban public program	274^b^	9535	5623	16,667		
Government rural public program	152	6340	4262	9056		
Uninsured population	83^c^	6937	5061	18,273		

*The Kruskal-Wallis test was used for comparisons

^a^8 responses were excluded from analysis because the information was incomplete or implausible

^b^1 response was excluded from analysis because the information was incomplete or implausible

^c^25 responses were excluded from analysis because the information was incomplete or implausible

### Relationship between cost of hospitalization and level of hospital

Two hundred and sixty-four inpatients (44.5 %) were treated in secondary hospitals and 329 (55.5 %) were treated in tertiary hospitals. The median total cost of one hospitalization per time per patient in RMB was significantly higher in tertiary hospitals than in secondary hospitals (9778 vs 6780) (*p* < 0.0001) (Table 5).

**Table 5:** Hospitalization expenses and level of hospital (in Renminbi)

	N	Mean cost	Standard deviation	Median cost	P25	P75	Z*	*P* value*
Secondary Hospitals	264	10,268	11,181	6780	4016	12,196	−5.7639	<0.0001
Tertiary Hospitals	329	14,687	16,599	9778	6075	17,633		

*The Wilcoxon Two-Sample Test was used for comparisons

### Hospitalization expenses in relation to disease stage

Inpatient costs were assessed by disease stage, with patients classified as “chronic HCV, no cirrhosis or HCC”, “chronic HCV plus cirrhosis” or “chronic HCV plus HCC”. The median total cost increased significantly with the severity of the disease stage, from 8112 RMB to 8399 RMB to 14,425 RMB (p = 0.0353) (Table 6).

**Table 6:** Inpatient costs by disease stage (in Renminbi)

	N	Median	P25	P75	*X* ^2^	P*
Chronic HCV, no cirrhosis or HCC	485	8112	5144	15,344	6.685	0.0353
Chronic HCV plus cirrhosis	95	8399	4746	15,004.0		
Chronic HCV plus HCC	13	14,425	14,114	25,995		

*The Kruskal-Wallis test was used for comparisons

### Outpatient expenses for antiviral therapy

Among the 404 outpatients who had received antiviral therapy, the following median costs were reported for a treatment duration of up to one year: 37,400 RMB for pegylated interferon-alpha (*N* = 197), 6798 RMB for interferon-alpha (N =185) and 377 RMB for ribavirin (*N* = 341) (Table 7).

**Table 7:** Outpatient antiviral therapy costs (in Renminbi)^a^

	N	Mean	Standard deviation	Median	P25	P75
Pegylated interferon-alpha	197	37,968	22,182	37,400	17,550	59,424
Interferon-alpha	185	7256	6397	6798	2790	9877
Ribavirin	341	643	1055	377	172	660

^a^The amount attributed to each antiviral drug was for up to one year of treatment

## Discussion

To our knowledge, this is the first study to examine medical costs associated with management of chronic HCV infection in mainland China. Although fairly high proportions of both inpatients and outpatients reported being covered by one of China’s three major types of government health insurance, 13 % of inpatients and 43 % of outpatients reported having no insurance. Among inpatients, the total median cost per hospitalization per patient was 8212 RMB. The category of expenditure with the highest median cost per hospitalization was Western medicine, followed by lab tests and Chinese medicine. The median cost per hospitalization was much higher for patients who had hepatocellular carcinoma than for those with less severe forms of liver disease. Additionally, outpatients reported high costs for antiviral drugs.

Beginning in the late 1990s, the Chinese government implemented a series of reforms for the purpose of making health care more affordable, but it is not clear whether the intended benifits are being realized [11, 12]. Our study of chronic HCV patients found that even while study participants reported utilizing all three major types of government health insurance, a high proportion of patients had no insurance of any kind. The impact of this situation on individual and household financial well-being may be devastating in light of the high costs associated with HCV disease.

The median cost per hospitalization for uninsured inpatients in our study was reported to be almost 7000 RMB. Although this study did not disaggregate outpatient antiviral therapy costs by insurance status, the fact that more than two in five outpatients were uninsured suggests that a considerable number of people paid out-of-pocket for pegylated interferon-alpha, interferon alpha and ribavirin. The median costs reported for these medicines were 37,400 RMB, 6798 RMB and 377 RMB respectively. To put these inpatient and outpatient costs in context, China had an annual per-capita income of 23,979 RMB in cities and towns in 2011, while the annual per-capita income for rural residents was 6977 RMB [13]. Thus it would be unsurprising for a single hospitalization to consume one-third of an uninsured urban HCV patient’s annual income or the entirety of an uninsured rural HCV patient’s annual income. The costs of the PEG-IFN/RBV and IFN/RBV regimens for outpatients are also far out of proportion to what the average Chinese person earns.

Insurance coverage gaps for HCV patients may also have grave implications for patient outcomes. In our study, the median inpatient cost per hospitalization for uninsured patients was 6937 RMB – slightly more than the median for patients covered by the government rural public insurance program. In contrast, median costs per hospitalization were more than 9500 RMB and more than 12,000 RMB for inpatients covered by the government employee insurance program and government urban public insurance program respectively. While this difference might be attributable to multiple reasons, one troubling possibility is that uninsured inpatients are receiving less of the health care services that they need because of perceived or actual inability to pay. The finding thus points to an important issue that warrants further attention from Chinese researchers and policy-makers. Research elsewhere has directly assessed the health consequences of a lack of insurance for HCV patients. A recent study by Younossi et al. reported that during the years 2005 to 2009, uninsured HCV patients in the United States had a 49 to 72 % greater chance of dying during a hospitalization than HCV patients who had insurance [14] .

Our analysis of the cost of hospitalization in relation to HCV disease stage indicated that a hospitalization for an HCV patient with hepatocellular carcinoma was typically far more costly than a hospitalization for an HCV patient who did not have HCC. Research from Canada and the United States also has found higher costs to be associated with more advanced HCV disease stage. A Canadian study that utilized modeling data for the estimated national population of HCV-infected people projected increasingly higher lifetime HCV costs for patients at more advanced disease stages [15]. In a large US cohort, chronic HCV patients with end-stage liver disease had far higher all-cause health care costs than chronic HCV patients with noncirrhotic liver disease or those with compensated cirrhosis. Approximately half of costs were HCV-related, with a higher proportion of HCV-related costs concentrated among patients with end-stage liver disease [16].

While this relationship between cost and disease stage is not unexpected, it bears emphasizing in light of the fact that HCV is a curable disease. The cost of providing treatment to chronic HCV patients in China may be well less than the cost of managing patients with untreated chronic HCV, and research is urgently needed to explore cost-benefit dynamics in the Chinese context. A US study quantified the direct economic burden of HCV infection and PEG-IFN/RBV treatment for HCV from 2002 to 2007, and found that PEG/IFN-RBV-treated patients had higher total direct medical costs than untreated HCV patients (US $28,547 vs. US $21,752; *P* < 0.001) [17]. Other research has found that despite the high cost of antiviral treatment, compared to discontinued therapy patients, chronic hepatitis C patients who completed interferon therapy and presumably had a higher rate of achieving sustained viral response were found to have lower levels of healthcare resource utilization and costs post-therapy [18]. Also, Federico et al. [19] showed that Canadian patients who had cleared the virus had the lowest time and out-of-pocket costs. Thus it seems feasible that increasing the use of antiviral therapy for HCV in China can reduce the overall economic burden in addition to improving health outcomes, and it is important for policy-makers to explore the potential for expansions in insurance coverage to contribute to overall financial gains in this regard.

This study has some limitations. First it is a cross-sectional investigation which does not reflect the dynamic changes in medical costs for all HCV patients. Second, the sample size is not large enough. A larger investigation needs to be conducted to acquire a more comprehensive dataset that can reliably guide health policy decision-making. Finally, there might be some recall bias in this study, such as the exact time of HCV diagnosis. Since we investigated outpatient medical cost within one year and per hospitalization cost, we consider it played a minor impact on the analysis of medical costs.

## Conclusions

For uninsured chronic HCV patients in China, inpatient and outpatient costs may be financially devastating. Chinese policy-makers need a better knowledge base in order to develop informed strategies for expanding insurance coverage. Research is needed on how different approaches to financing HCV treatment and care might improve health outcomes as well as achieve cost savings by enabling more people to be cured of HCV.

## Abbreviations

AASLD: American association for the study of liver diseases
DAAs: direct-acting antivirals
HCC: hepatocellular carcinoma
HCV: hepatitis C virus
PEG-IFN: peg-interferon
RBV: Ribavirin
RMB: Renminbi
